# Possible Mechanisms of Lymphopenia in Severe Tuberculosis

**DOI:** 10.3390/microorganisms11112640

**Published:** 2023-10-27

**Authors:** Fei Li, Dandan Chen, Qingqing Zeng, Yunjie Du

**Affiliations:** Institute of Pathogen Biology, School of Basic Medical Sciences, Lanzhou University, Lanzhou 730000, China; 220220912200@lzu.edu.cn (D.C.); zengqq21@lzu.edu.cn (Q.Z.); 120220905730@lzu.edu.cn (Y.D.)

**Keywords:** tuberculosis, lymphopenia, hematopoiesis, chronic infection, apoptosis

## Abstract

Tuberculosis (TB) is a chronic infectious disease caused by *Mycobacterium tuberculosis* (*M. tuberculosis*). In lymphopenia, T cells are typically characterized by progressive loss and a decrease in their count results. Lymphopenia can hinder immune responses and lead to systemic immunosuppression, which is strongly associated with mortality. Lymphopenia is a significant immunological abnormality in the majority of patients with severe and advanced TB, and its severity is linked to disease outcomes. However, the underlying mechanism remains unclear. Currently, the research on the pathogenesis of lymphopenia during *M. tuberculosis* infection mainly focuses on how it affects lymphocyte production, survival, or tissue redistribution. This includes impairing hematopoiesis, inhibiting T-cell proliferation, and inducing lymphocyte apoptosis. In this study, we have compiled the latest research on the possible mechanisms that may cause lymphopenia during *M. tuberculosis* infection. Lymphopenia may have serious consequences in severe TB patients. Additionally, we discuss in detail potential intervention strategies to prevent lymphopenia, which could help understand TB immunopathogenesis and achieve the goal of preventing and treating severe TB.

## 1. Introduction

Tuberculosis (TB) is a major infectious disease caused by *Mycobacterium tuberculosis* (*M. tuberculosis*) [1]. TB is an ancient disease that originated in 17th–18th century Europe and is commonly referred to as the “white plague”, with an almost 100% infection rate and a mortality rate of 25% [2]. However, with the development of anti-TB drugs, improved sanitation, and better living conditions, the incidence and mortality of TB have significantly decreased. However, since the 1990s, due to the emergence of drug-resistant TB, the use of immunosuppressants, poverty, and population mobility, TB has resurged. According to the World Health Organization (WHO) Global TB Report 2022, about a quarter of the world’s population is infected with *M. tuberculosis*, there are about 10.6 million new TB cases worldwide each year, and 1.6 million people die from TB [3]. China is a high-TB-burden country, with the number of TB cases second only to India and Indonesia [3]. Therefore, TB remains a global public health problem.

Antigen-specific T/B lymphocytes play a critical role in the host response to *M. tuberculosis* infection. *M. tuberculosis* infection activates the body’s protective Th1-type cell-mediated immune responses by secreting cytokines such as IFN-γ and IL-2, which further activate the macrophage system to effectively control and eradicate *M. tuberculosis* [4,5]. Although Th1 cells play a major role in controlling intracellular pathogen infection, Th2 cells and CD8^+^ T cells are also important in combating *M. tuberculosis* infection. Th2 cells mainly release IL-4 and IL-10, participate in the activation and proliferation of B cells, and assist in the regulation of humoral immunity [6]. Upon infection, CD8^+^ T cells are also activated and differentiate into cytotoxic T lymphocytes (CTLs), which dissolve macrophages that have lost immune activity by releasing perforin and granulysin, eventually eliminating *M. tuberculosis* [7].

Severe forms of TB include military TB, multidrug-resistant tuberculosis (MDR-TB), and TB meningitis [8]. Some severe TB patients even need to be admitted to the intensive care unit (ICU) [9]. Severe TB can cause various complications, including severe weight loss, anemia, and lymphopenia, ultimately leading to death [10,11,12]. The mortality rates for these severe forms range from 15.5% to 65.9% [13,14]. Lymphopenia is a common sign in clinical patients with advanced and severe TB, but its underlying causes are still unclear. Therefore, it is necessary to investigate new mechanisms of lymphopenia to explore potential therapeutic targets. 

## 2. Lymphopenia Is Common in TB Patients

The cellular immune response, which is mediated by T lymphocytes, plays a crucial role in anti-TB immunity. To understand the immune response following *M. tuberculosis* infection, it is important to examine the phenotypes and function of T lymphocytes in human peripheral blood [15]. T cell counts are known to change in response to *M. tuberculosis* infection. Severe TB patients typically experience a decrease in the number of CD4^+^ T cells [16] along with an increase in the expression of immune checkpoint molecules and a decrease in function [17]. Additionally, both CD4^+^ and CD8^+^ T cell counts are decreased in active TB patients [15,18]. Lymphopenia is defined as lymphocyte percentage (the percentage of lymphocyte count to leukocyte count) < 20% or absolute lymphocyte count < 10^9^ cells/L [10,19]. Specifically, lymphopenia was found in 46% of untreated pulmonary TB patients [20] and 76.92% of patients with miliary TB [10], revealing that lymphopenia may be a prominent feature of severe TB infection [21]. More recently, CD4^+^ T cell lymphopenia has emerged as a significant risk factor in TB-infected patients [22]. Therefore, lymphopenia is a common occurrence in TB patients.

Furthermore, the degree of lymphopenia is associated with disease severity [23,24]. The more severe the disease, the lower the number of CD4^+^ T cell counts. The counts of leukocytes and the ratios of lymphocytes and monocytes during TB therapy are used clinically to evaluate the efficacy of treatment [25]. However, the pathogenesis of CD4^+^ T cell lymphopenia still lacks in-depth research, and further studies are needed to investigate the mechanisms of lymphopenia during severe *M. tuberculosis* infection and the possible treatment implications for these patients. 

## 3. The Possible Mechanisms That Cause Lymphopenia

**Figure 1:**
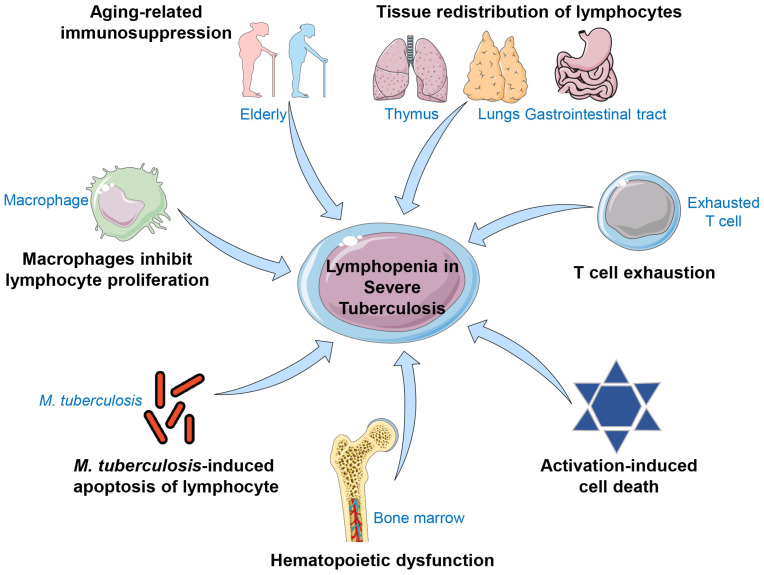
The possible mechanisms that may lead to lymphopenia in severe TB patients. Lymphopenia is a common sign in severe TB patients and is affected by various factors, ranging from impaired production to function and survival. The detailed mechanisms consist of macrophages inhibiting lymphocyte proliferation, *M. tuberculosis*-induced T lymphocyte apoptosis, hematopoietic dysfunction, activation-induced cell death, T cell exhaustion, tissue redistribution of lymphocytes, and aging-related immunosuppression.

The various mechanisms mentioned here may work together and overlap in some instances, leading to lymphopenia in TB patients. The potential mechanisms of *M. tuberculosis*-induced lymphopenia are discussed below (Figure 1). However, further in vitro and in vivo studies are required to determine the importance of each component in the occurrence of lymphopenia. 

### 3.1. Macrophages Inhibit Lymphocyte Proliferation

*M. tuberculosis* is an intracellular pathogen that resides predominantly within macrophages, which serve as the first line of host defense against bacterial infections. After *M. tuberculosis* infects the body, macrophages recognize the corresponding pathogen-associated molecular patterns (PAMPs) through pattern recognition receptors (PRRs) such as Toll-like receptors (TLRs) on the surface, thereby activating macrophages. After a series of signal transductions, activated macrophages generate a large number of cytokines, which further activate T cells to trigger an adaptive immune response and eradicate pathogens. In addition, IFN-γ has a central role in host defense against *M. tuberculosis* by activating macrophages [26]. However, activated macrophages can also inhibit T cell proliferation through Stat6-dependent PD-L2 expression [27]. 

The immunomodulatory properties of alveolar macrophages are relatively selective. Alveolar macrophages selectively inhibit the proliferation of T cells but permit T cell activation and effector functions such as cytokine secretion within the lungs [28,29]. Upham et al. demonstrated that human alveolar macrophages can inhibit T-cell proliferation in vitro [30]. Since IL-2 interacting with its specific receptor can trigger T cell proliferation, the mechanism may be that alveolar macrophages block the protein tyrosine’s phosphorylation related to IL-2 receptors, interfering with the normal function of IL-2 receptors, and ultimately inhibiting T cell proliferation [28].

The cytokines secreted by macrophages can also inhibit the proliferation of T cells and affect the host’s immunity against *M. tuberculosis*. *M. tuberculosis* can induce macrophages, T cells, and dendritic cells to secrete cytokines, such as tumor necrosis factor-α (TNF-α), which plays a pivotal role in T-cell-mediated adaptive immunity [31]. In addition, TNF-α is also a negative regulator that can inhibit T cell proliferation and reduce the T cell activation, thereby regulating the immune response [28,32]. 

### 3.2. M. tuberculosis-Induced Apoptosis of T Lymphocyte

Lymphopenia is also driven by lymphocyte apoptosis [33]. Lymphocyte apoptosis, an important immune regulatory mechanism, is linked to cell-mediated effector dysfunction in infectious diseases [34]. The increased apoptosis of CD4^+^ T lymphocytes in peripheral blood is related to lymphopenia. The apoptosis of Th1 lymphocytes leads to disease progression, while the apoptosis of macrophages favors disease protection [35]. T-cell apoptosis can significantly reduce the number of cells, thereby weakening the body’s anti-TB immune response, leading to the occurrence and development of TB [36]. The apoptosis rate of CD4^+^ T cells in peripheral blood from patients with sputum-positive pulmonary TB is significantly higher than that of patients with sputum-negative pulmonary TB and healthy controls. Moreover, immunodeficiency in patients with acutely progressive TB is associated with the apoptosis of immunocompetent cells, and the main clinical marker of apoptosis is the significant reduction in lymphocytes (4–10%) [37]. Virulent *Mycobacterium avium* (*M. avium*) infection also causes progressive severe lymphopenia due to increased apoptosis rates [38]. The apoptosis of CD4^+^ T cells and CD8^+^ T cells during *M. avium* infection is dependent on Fas/Fas ligand (FasL) and Bcl-2-sensitive pathways, respectively [39]. The intervention of certain factors that cause apoptosis is expected to bring new hope and breakthroughs in TB therapy. 

*M. tuberculosis* antigens can directly cause T lymphocyte apoptosis. Studies have demonstrated that *M. tuberculosis* heat shock protein (HSP)-70, 65, and 16 could induce the apoptosis of peripheral blood monocytes and CD4^+^ and CD8^+^ lymphocytes in TB patients. The apoptosis in TB may be associated with the levels of TNF-α and NO, which are controlled by *SLC11A1* (formerly *NRAMP*) [40]. The purified protein derivative of *M. tuberculosis* (Mtb-PPD) can induce T cell apoptosis by exploiting the Fas/FasL system [41]. Furthermore, *M. tuberculosis* antigen MPT64 could induce the apoptosis of EL4 lymphoma cells in a dose-dependent manner, which may be related to the elevated levels of IL-4, transforming growth factor-β (TGF-β), and Fas [42]. When CD4^+^ and γδ T cells from TB patients are cultured with *M. tuberculosis*, T cell apoptosis increases [36]. It has also been shown that the H37Rv strain causes a progressive increase in apoptotic Th1 lymphocytes mediated by Fas/FasL interaction [35]. Besides that, macrophages infected with *M. tuberculosis* may induce specific and non-specific T-cell apoptosis by nitric oxide (NO) and TNF-α [43]. 

The alteration of cytokines in TB patients is linked to lymphocyte apoptosis. A clinical study has shown a positive correlation between the apoptotic rate of CD4^+^ T cells in TB patients and the secretion levels of IFN-γ, IL-4, TGF-β, and TNF-α [44]. IFN-γ is thought to be the cause of T cell death in mycobacterial infections [45]. IFN-γ can stimulate macrophages to produce NO, which induces apoptosis in T cells, thus IFN-γ causes apoptosis of activated CD4^+^ T cells during mycobacterial infection by activating macrophages and NO [45]. IFN-γ-dependent apoptosis is observed in T cells. Some studies have also demonstrated that Fas and cleaved caspase 8 are involved in IFN-γ-mediated apoptosis [46]. Meanwhile, TNF-α signaling is also an important part of T cell-induced apoptosis by recruiting FADD and caspase-8 to the receptor complex II [47]. The response of peripheral blood lymphocytes from healthy donors to sonicated *M. tuberculosis* antigens shows an increase in IL-4 gene expression and apoptosis, while inhibiting IL-4 expression could reduce TNF-α-mediated lymphocyte apoptosis [34]. The mechanism may be that *M. tuberculosis*-activated lymphocytes induce IL-4 expression, thereby promoting the CD30^+^ population, which reduces TNF receptor-associated factor 2 (TRAF2) expression and enhances TNF-α-mediated apoptosis in lymphocytes [34]. These data suggest that the reduction of peripheral blood lymphocytes in TB patients may be mediated by cytokines, but further studies are needed to understand the mechanism.

In addition, upregulated perforin/granzyme expression may also be associated with the increased apoptosis of T cells in TB patients by synergizing with other genes, including caspase-3 and caspase-10 [48]. Effector CD8^+^ T cells from patients with severe TB also overexpress a variety of cytotoxic genes, like GZMA, GZMB, etc. Likewise, significantly increased expression of caspase-3 is detected in severe TB patients [21]. Moreover, it is reported that the pro-apoptotic gene X-linked inhibitor of apoptosis-associated factor 1 (XAF-1) [49] cooperates with caspase-3, TP53, and BCL2L11 to induce cell apoptosis. TNF can activate XAF1, which acts as an alternative pathway for TNF-associated apoptosis [50]. XAF1 also promotes TP53-related apoptosis by post-translational modification [21]. Furthermore, the immune checkpoint molecule Tim-3/Galectin-9 axis plays a critical role in inhibiting Th1 cell immunity and can lead to the apoptosis of Th1 cells by activating caspase-1 [51,52]. Using anti-Tim-3 antibodies to blockade Tim-3 could decrease the severity of the inflammatory responses and reduce CD8^+^ T cell apoptosis [53].

### 3.3. M. tuberculosis-Mediated Bone Marrow Hematopoietic Dysfunction

Chronic infections significantly affect the expansion and differentiation of bone marrow hematopoietic cells [54,55], which further influence the production of lymphocytes. Hematopoietic stem cells (HSCs), a population of stem cells, can give rise to all lineages of blood cells [56,57]. Infection-specific changes in the hematopoietic system may limit or promote the production of specific lineages, thereby centrally affecting the immune response. For instance, *M. tuberculosis* reprograms HSCs to limit myelopoiesis and impairs the development of protective trained immunity to *M. tuberculosis* infection in a type I interferon (IFN-I)-dependent manner [58]. Repeated infections of mice with *M. avium* depleted the pool of HSCs and made mice develop pancytopenia [59]. Moreover, *Mycobacterium bovis* Bacillus Calmette–Guérin (BCG) can train HSCs to enhance myelopoiesis at the expense of lymphopoiesis via the activation of IFN-II (IFN-γ) signaling and generate protective innate immunity against *M. tuberculosis* infection [60,61]. However, severe TB causes hematopoietic dysfunction and immune exhaustion due to continuous *M. tuberculosis* stimulation [10,62]. 

Some inflammatory cytokines are critical for regulating hematopoiesis and determining the differentiation fate of bone marrow hematopoietic cells. Among them, IFN-γ impairs the proliferation and reconstitution of HSCs and affects the differentiation from long-term HSCs (LT-HSCs) to short-term HSCs (ST-HSCs) [63,64]. Furthermore, chronic infection with *M. avium* and persistent *M. tuberculosis* antigen stimulation deplete lymphoid commitment due to persistent IFN-γ stimulation and induce lymphopenia [10]. IFN-γ promotes myelopoiesis by upregulating the expression of Batf2 [59]. Similar to IFN-γ, TNF-α also promotes myeloid differentiation [65]. Unlike IFN-γ, IL-7 is a crucial cytokine in lymphopoiesis and T cell homeostasis [66,67], which is essential for normal thymopoiesis [68], inhibiting apoptosis and promoting T cell survival [69]. IL-7 can rescue B-lymphopoiesis by driving the proliferation and expansion of B-cell progenitors [70,71]. Interestingly, a molecule called chromodomain helicase DNA-binding protein 4 (CHD4) is required for IL-7 receptor signaling and is also essential for transcriptional repression and lineage commitment during B lymphopoiesis [72]. Similarly, IL-2 can also enhance lymphoid differentiation, thereby reversing impaired hemopoiesis and lymphopenia [10,12]. As hematopoietic cells are the source of immune cells, the occurrence of lymphopenia is largely attributed to hematopoietic dysfunction.

Chemokines also play a critical role in the process of hematopoiesis, thereby affecting the production of T cells [73]. During chronic infection, the expression of chemokine CXCL10 can be induced by innate immune cell cytokines IFN-α/β as well as adaptive immune stimuli IFN-γ. CXCL10 is upregulated in TB patients and could inhibit the development of hematopoietic precursors [74], which may contribute to TB-associated lymphopenia. In the hematopoietic system, CXCR4 binds to its chemokine ligand, CXCL12, to mediate leukocyte transport, distribution, survival, activation, and proliferation. CXCR4 has been reported to help thymic progenitors homing from bone marrow to the thymus and emerges as a promising proposed TB target [75].

Post-translational modifications of proteins, particularly ubiquitination, play a significant role in the hematopoietic process and are closely associated with lymphopenia. Most E3 ubiquitin ligases have a negative impact on the development and function of HSCs. The E3 ubiquitin ligase c-Cbl is a novel negative regulator of HSC development and function. When c-Cbl is absent, it increases the number of HSCs and enhances the capacity to rebuild the hematopoietic system [76]. C-Cbl deficiency can lead to a decline in lymphocyte development and function in aging individuals [77]. Similarly, another E3 ubiquitin ligase Itch can also negatively regulate the development and function of HSCs, and its deficiency can also result in hematopoietic abnormalities [78]. The E3 ubiquitin ligase Fbw7 plays a crucial role in regulating HSC quiescence and self-renewal. Fbw7 is expressed in quiescent HSCs. Once it loses its self-renewal ability, its expression is downregulated. Deletion of Fbw7 in bone marrow stem cells and progenitors results in decreased hematopoietic progenitors and impaired self-renewal ability [79]. Recently, studies have demonstrated that the absence of ubiquitin editing enzyme A20 can lead to the overexpression of IFN-γ, leading to abnormal differentiation of lymphoid cells and promoting the differentiation into the myeloid lineage, ultimately causing lymphopenia [80]. 

### 3.4. T Cell Exhaustion

In many chronic infections and cancer, persistent antigen stimulation can lead to T cell exhaustion, which is a state of T cell dysfunction characterized by progressive loss of effector function and memory T cell potential as well as high and sustained expression of inhibitory immune checkpoint receptors [81,82]. In severe TB, the levels of IFN-γ and TNF from activated CD4^+^ and CD8^+^ T cells are lower, while the expression of multiple inhibitory molecules, including PD-1, Tim-3, etc., are high. These data suggest that T cells become dysfunctional in severe TB and fail to control *M. tuberculosis* infection [21]. In addition, T cell exhaustion is accompanied by alteration in transcription factor expression [83,84], and exhausted T cells have distinct transcriptional profiles, which include a subset of transcription factors, like positive regulatory domain 1 gene (PRDM1)/B lymphocyte-induced maturation protein-1 (Blimp-1) [85], basic leucine zipper ATF-like transcription factor (BATF) [86], and nuclear factor of activated T cells (NFAT) [87]. As such, significantly elevated expression of PRDM1 is found in severe TB patients compared with healthy donors [21]. Furthermore, the cells that lack CD28 expression are considered severely exhausted, and CD28-exhausted CD4^+^ T cells are linked to lymphocyte counts and poor prognosis [88]. 

### 3.5. Tissue Redistribution of Lymphocytes

*M. tuberculosis* infection could induce lymphocyte trafficking to secondary lymphoid tissues [89]. The decrease in blood lymphocytes may be due to T cells redistributing to the lungs and gastrointestinal tract. Chemokines and chemotactic cytokines can stimulate the migration of T lymphocytes to the lung [90]. TNF coordinates the induction of chemokines to recruit leukocytes to form granulomas in TB [91]. It has been suggested that the rapid increase in lymphocyte count during recovery is not a result of newly produced lymphocytes in the thymus but rather the result of lymphocyte recirculation between organs and peripheral blood.

Cell migration pathways contribute to the reduction of lymphocytes. Migration-associated genes like CXCL10, CXCL8, CCL3, etc., are upregulated in CD8^+^ T cells from severe TB patients, indicating that T cells display a significant activation of migration. Consistent with the results above, NK cells from severe TB patients also show high migration and death, which may be associated with the reduction in the NK population [21].

### 3.6. Aging-Related Immunosuppression

The aging population faces a significantly increased risk of infection and cancer. Immunosenescence can affect innate immunity and adaptive immunity. Aging is associated with a decrease in the number of hematopoietic precursors and the degeneration of the thymus, both of which lead to the reduced ability of newborn T cells. Therefore, the functional capacity of T cells is affected by aging, with a limited available T cell pool and a reduced number of naïve T cells [92,93]. Thymus activity persists into adulthood, and as the thymus shrinks, its function gradually decreases with age. The underlying mechanisms causing thymic atrophy still need to be investigated. Additionally, several age-related comorbidities like obesity, diabetes, malnutrition, chronic respiratory diseases, and cancer can increase the risk of developing active TB [94]. 

Various agents for bone marrow transplantation and aging research represent promising strategies for combating T-cell deficiencies in the aging population. Preclinical and clinical studies on the effects of keratinocyte growth factor, sex steroid ablation, and the cytokine IL-7 on T cell reconstitution suggest that these strategies can be utilized to alleviate the effects of T cell deficiencies and enhance T cell reconstitution in the elderly [95]. 

### 3.7. Activation-Induced Cell Death in Lymphocytes

Activation-induced cell death (AICD) in T lymphocytes has been recognized as one of the mechanisms of lymphopenia. T cells undergo AICD during contraction, which is triggered by the death receptor and results in apoptosis [96,97]. AICD is mediated by the Fas/FasL pathway [98]. Fas and FasL are a pair of receptors/ligands that are critically involved in lymphocyte homeostasis. The interaction between Fas and FasL triggers apoptosis in Fas-expressing cells [99]. The levels of Fas in PBMC are associated with the severity of TB [100]. The FasL surface and mRNA expression in peripheral blood mononuclear cells (PBMCs) from TB patients are higher compared to controls. Additionally, neutralizing FasL can eliminate T-cell apoptosis [44]. Furthermore, the loss of γδT cells is a consequence of *M. tuberculosis* antigen-mediated AICD through the interaction between Fas and FasL [101]. 

Except for FasL, AICD is also mediated by other receptor/ligand pathways such as TNF and TNF-receptor-related apoptosis-inducing ligand (TRAIL) [102]. In a mouse model of *M. avium* infection, both CD4^+^ and CD8^+^ T cells suffered a higher rate of apoptosis. Among them, low expression of Bcl-2 and excessive expression of TNF-α, TGF-β, and FasL are involved in the apoptosis of CD4^+^ T cells [44]. This suggests that there may be differences in the apoptotic pathways of CD4^+^ and CD8^+^ T cells, which are closely related to the expression levels of TNF receptors and Bcl-2 [39].

### 3.8. Myeloid-Deprived Suppressor Cells 

Myeloid-deprived suppressor cells (MDSCs) are an immature population of myeloid cells that enter a pathological activation state and can suppress T-cell responses [103]. The levels of monocytic MDSCs are increased significantly in the peripheral blood of patients with severe TB [21,104]. In mycobacterial infections, such as *M. tuberculosis*, *Mycobacterium smegmatis* (*M. smeg*), and BCG, MDSC is induced [105]. Due to MDSC immunosuppressive activity, T cell functions like cytokine production, T cell activation, and modulation of T cell trafficking are inhibited [106], consequently decreasing T cell counts. Human MDSC upregulates PD-L1 during in vitro mycobacterial infection and utilizes this checkpoint molecule to limit T cell proliferation [107]. MDSC may promote TB reactivation by exacerbating the immunosuppressive effects of therapies such as anti-TNF drugs, and the deficiency of TNF-α is associated with T cell immunosuppression [108].

## 4. Potential Immunotherapies

As discussed above, substantial evidence supports a significant correlation between *M. tuberculosis*-induced lymphopenia and lymphocyte production, function, and survival. Therefore, targeting the specific TB immune profiles, such as reducing apoptosis or enhancing lymphopoiesis, is a promising treatment strategy to rescue lymphopenia and also compensate for the lymphocyte counts in severe TB. Therapy that enhances lymphopoiesis includes IL-7/IL-2 therapy. To inhibit apoptosis, strategies such as TNF-α or TGF-β antibodies can be employed (Table 1). 

### 4.1. Reducing Apoptosis

Targeting the inhibition of apoptosis may prevent lymphopenia and compensate for the low number of lymphocytes in severe TB patients. FasL-mediated apoptosis plays a critical role in lymphocyte homeostasis and results in lymphopenia. Therefore, inhibiting the interaction between Fas and FasL could prevent the depletion of lymphocytes [109]. Fas–FasL interaction also induces T-cell apoptosis in AICD. Furthermore, the apoptosis of CD4^+^ T cells is closely related to the low expression of Bcl-2 and excessive expression of TNF-α, TGF-β, and FasL [44]. Thus, defective Fas or Bcl-2 overexpression protects T cells from TNF receptor-related apoptosis. Moreover, the use of IFN-γ, TNF-α, or TGF-β antibodies can reduce spontaneous or *M. tuberculosis*-induced T cell apoptosis [34,44,46]. Biologically, taurine is a semi-essential amino acid synthesized from methionine and cysteine. Taurine attenuates CD3/IL-2-induced T cell apoptosis in AICD and augments the immunotherapeutic potential of IL-2 [110]. Several strategies involved in inhibiting lymphocyte egress from secondary lymphoid tissues may also reverse lymphopenia.

However, the drawbacks of these treatment strategies cannot be ignored. For instance, a subset of cancers originating from the lymphatic system displays defective Fas/FasL-mediated apoptosis, rendering them resistant to chemotherapy [111]. Bcl-2 is commonly overexpressed in various types of cancers, especially in lymphoma. Therefore, treatment with Bcl-2 overexpression may be linked to therapeutic resistance and poor prognosis [112]. Considering that TNF-α is a cytokine involved in immune responses, blocking TNF-α may pose a unique risk of latent tuberculosis infection (LTBI) reactivation and new infections due to impaired effector immune responses [113]. Additionally, TGF-β plays a crucial role in the expansion of Th17 cells against mycobacterial infection [114]. Consequently, the extent to which the TGF-β blockade interferes with immune response in reducing apoptosis therapy is still unclear and requires further investigation.

Furthermore, there exists another view about the impact of T cell apoptosis, which suggests that T cell apoptosis might serve as a host regulatory mechanism to restrict excessive inflammation and prevent tissue damage, rather than an *M. tuberculosis*-induced immune deviation [115]. In line with this, certain studies have shown that mesenchymal stem cells (MSCs) can induce activated T cell apoptosis by producing NO, thereby weakening the delayed-type hypersensitivity (DTH) response [116]. Hence, additional research is required to ascertain the relevance of apoptosis in the development of TB and to uncover the underlying mechanisms. 

### 4.2. Targeting Lymphocyte Generation 

Some inflammatory cytokines can regulate the homeostasis and differentiation of hematopoietic cells [117]. Cytokines IL-7, IL-2, and stem cell factor (SCF) are proposed therapies for enhancing lymphopoiesis [118]. Infection with *M. tuberculosis* could impact the differentiation of hematopoietic cells, leading to a decrease in lymphocyte production [10,60]. In addition, the differentiation of hematopoietic cells is tightly regulated by lineage-determining transcription factors [119]. Among the transcription factors, interferon regulatory factor-8 (IRF8) and Batf2 tend to promote myeloid differentiation [59], while GATA2 and NOTCH1 are critical for T cell commitment [12,120]. Moreover, interferon regulatory factor-4 (IRF4) or paired box-5 (Pax5) have been associated with lymphopoiesis [121,122]. Therefore, upregulating the expressions of transcription factors, such as NOTCH1 and GATA2, involved in T-cell lineage commitment and down-regulating IFR8 and Batf2 participation in myeloid differentiation are seen as possibly useful strategies for enhancing lymphopoiesis and reversing lymphopenia.

Maintaining the homeostasis of hemopoiesis is crucial for establishing an effective immune response against TB [12]. When homeostasis is disrupted, it can impact the differentiation of HSCs. The objective of enhancing lymphopoiesis is to restore a steady state of hematopoiesis. Insufficient production of lymphocytes can result in a poor therapeutic effect, while excessive production can lead to side effects such as lymphocytosis. Therefore, immunotherapy needs to investigate the timing and dosage to achieve the optimal therapeutic outcome.

**Table 1:** Promising therapeutic strategies for severe TB.

Strategy	Major Changes	Intervention Methods	Intervention Mechanism
Reducing apoptosis	FasL ↑	Fas antibody [109]	Attenuates CD3/IL-2-induced T cell apoptosis
	Bcl-2 ↓	Bcl-2 overexpression [44]	Protects T cells from TNF receptor-related apoptosis
	IFN-γ, IL-4, TNF-α, or TGF-β ↑	IFN-γ antibody [46], IL-4 antibody [34], TNF-α antibody [34], TGF-β antibody [44]	Reduces the cytokines-mediated lymphocyte apoptosis
Targeting lymphocyte generation	IL-7, IL-2, SCF, NOTCH1, GATA2, IRF4, Pax5 ↓	IL-7, IL-2, SCF [118], NOTCH1 enhancer, GATA2 enhancer [12,120], IRF4 enhancer, and Pax5 enhancer [121,122]	Promote lymphopoiesis
	IFR8, Batf2 ↑	IFR8 and Batf2 inhibitors [59]	Reduces myeloid differentiation to promote lymphopoiesis

Notes: The arrow ↑ indicates up-regulated gene expression, while the arrow ↓ indicates down-regulated gene expression.

## 5. Conclusions and Perspectives

Tuberculosis is a chronic wasting disease, and its immune response level changes with the development of the disease. Peripheral blood lymphocytes are important cells involved in the body’s anti-TB immunity. Their quantity and function can be used to evaluate the body’s immune function and determine whether the host is infected by *M. tuberculosis*, which is closely related to the clinical severity of the disease. Lymphopenia often occurs in TB patients. Multiple mechanisms may be involved in the occurrence of lymphopenia by affecting lymphocyte production, function, and survival. The main mechanisms include the inhibition of lymphocyte proliferation by macrophages, inducing T lymphocyte apoptosis, and bone marrow hematopoietic dysfunction. 

Lymphopenia may have serious consequences in severe TB patients. Due to the crucial role of *M. tuberculosis*-specific T cell-mediated immune responses in bacterial elimination, lymphopenia can cause bacterial persistence, replication, and tissue damage. Additionally, destroying a large number of lymphocytes can lead to general immunosuppression in TB patients. In accordance with COVID-19, lymphopenia also plays a key role in TB pathogenesis. In lymphopenia, the number of Treg cells (as the main regulator of immune responses, exhibiting immunosuppressive function) also decreases, thereby strengthening excessive inflammatory responses. Collectively, lymphopenia may lead to delayed elimination of *M. tuberculosis*, unrestricted Th1 response, uncontrolled cytokine production, and even cytokine storms, ultimately leading to multiple organ failure and death. Therefore, eliminating the factors that cause lymphopenia may be an effective therapeutic strategy for severe TB. However, further investigation on the pathogenesis of lymphopenia is still necessary to understand the interaction mechanism between *M. tuberculosis* and hosts. Conducting relevant studies will offer new insights for the prevention and treatment of severe TB.

## Data Availability

Data sharing not applicable.

## Abbreviations

TB: Tuberculosis; *M. tuberculosis*: *Mycobacterium tuberculosis*; MDR-TB: multidrug-resistant tuberculosis; HIV: human immunodeficiency virus; PAMP: pathogen-associated molecular pattern; PRR: pattern recognition receptor; TLR: Toll-like receptor; *M. avium*: *Mycobacterium avium*; TGF-β: transforming growth factor-β; NO: nitric oxide; XAF-1: X-linked inhibitor of apoptosis-associated factor 1; HSCs: hematopoietic stem cells; IFN-I: I interferon; BCG: *Mycobacterium bovis* Bacillus Calmette–Guérin; CHD4: Chromodomain helicase DNA-binding protein 4; PRDM1: positive regulatory domain 1; Blimp-1: B lymphocyte-induced maturation protein-1; BATF: basic leucine zipper ATF-like transcription factor; NFAT: nuclear factor of activated T cells; AICD: activation-induced cell death; PBMC: peripheral blood mononuclear cell; TRAIL: TNF-receptor-related apoptosis-inducing ligand; MDSC: myeloid-deprived suppressor cell; *M. smeg*: *Mycobacterium smegmatis*; MSCs: mesenchymal stem cells; DTH: delayed-type hypersensitivity; SCF: stem cell factor; IRF8: interferon regulatory factor-8; IRF4: interferon regulatory factor-4; Pax5: paired box-5.
